# Space-time clustering analyses of childhood acute lymphoblastic leukaemia by immunophenotype

**DOI:** 10.1038/sj.bjc.6600498

**Published:** 2002-08-27

**Authors:** R J Q McNally, F E Alexander, J M Birch

**Affiliations:** Cancer Research UK Paediatric & Familial Cancer Research Group, Stancliffe, Central Manchester and Manchester Children's University Hospitals NHS Trust, Hospital Road, Manchester M27 4HA, UK; Department of Public Health Sciences, The University of Edinburgh Medical School, Teviot Place, Edinburgh EH8 9AG, UK

**Keywords:** acute lymphoblastic leukaemia, children, aetiology, infection, immunophenotype, space-time clustering

## Abstract

Space-time clustering analyses of acute lymphoblastic leukaemia in children, by immunophenotype, were carried out using a population-based registry. Significant evidence was found of space-time clustering for cases of the precursor B-cell sub-type, in the childhood peak, based on time and location at birth.

*British Journal of Cancer* (2002) **87**, 513–515. doi:10.1038/sj.bjc.6600498 www.bjcancer.com

© 2002 Cancer Research UK

## 

We have previously reported significant evidence of space-time clustering in childhood leukaemia, diagnosed during 1954–1985, and based on place of birth and time of diagnosis, particularly among cases of acute lymphoblastic leukaemia [ALL] aged 0–4 years (Birch *et al*, 2000). The finding of space-time clustering is consistent with the involvement of infectious agents in the aetiology. There have been several hypotheses, supported by indirect epidemiological evidence, proposing such a role for infections. Kinlen (1988) demonstrated that situations of unusual rural population mixing, which promoted contacts between susceptible and infected individuals, are associated with excesses of ALL, whilst Greaves (1988) has proposed that delayed exposure to common infections would lead to a greater chance of the occurrence of precursor B-cell (common) ALL. Smith (1997) suggested that the relevant infectious exposures occur *in utero*. Our previous findings were consistent with Greaves’ model and we hypothesised that the clustering cases would mainly involve precursor B-cell ALL. It has not been possible previously to test this hypothesis directly, because no population-based data on ALL, by immunophenotype, were available over a sufficiently long time period. We have now been able to perform such an analysis, using cases of ALL included in the Manchester Children's Tumour Registry (MCTR), during 1980–2001, for which full data on immunophenotype are available. The aim of this new study was to test the prediction that space-time clustering is concentrated among cases of the precursor B-cell sub-type.

## PATIENTS AND METHODS

All cases, diagnosed with ALL between the 1st January 1980 and 31st December 2001, and registered by the MCTR were analysed. Ordnance Survey [OS] eight-digit grid references were allocated to each case with respect to addresses at time of birth and diagnosis, locating each address to within 0.1 kilometre. The following diagnostic groups were specified a priori for analysis: (1) all precursor B-cell (common) ALL; (2) precursor B-cell ALL, aged 18–54 months; (3) all non-precursor B-cell ALL; (4) non-precursor B-cell ALL, aged 18–54 months; (5) total ALL.

There are four possible space-time interactions between: (1) times and places of diagnosis; (2) times and places of birth; (3) time of diagnosis and place of birth; and (4) time of birth and place of diagnosis. The interpretation of these interactions will depend on the extent of migration between birth and diagnosis among cases (Birch *et al*, 2000).

Knox (1964) tests were applied to the data with thresholds fixed, a priori, as: close in space, less than 5 km, and close in time, less than 1 year apart. One-sided tests were used to detect a significant interaction. The strength of interactions (S) was indicated by calculating ((O−E)/E)×100 counts of pairs which are close in space and close in time (O, observed number of close pairs, and E, expected number of close pairs). To adjust for the effects of different population densities, the tests were repeated replacing geographical distance thresholds by distance to the Nth nearest neighbour, using all locations of all the cases in the data set except addresses for the same child at a different time. N was chosen such that the mean distance was 5 km (*N*=40 for birth locations and 41 for diagnosis locations).

As previously discussed, two problems are apparent with the Knox test: boundary problems and the arbitrariness of the thresholds chosen (Mcnally *et al*, 2002). To overcome these a second order procedure based on K-functions (Diggle *et al*, 1995) is used, employing both geographical distance and nearest neighbour (NN) approaches as described above.

Data were also analysed by examining clustering pairs which contained at least one male case (‘male: any’) and clustering pairs which contained at least one female case (‘female: any’). Using internal methods, addresses were classified as being located in a more densely populated area, or a less densely populated area (Mcnally *et al*, 2002). Analysis by population density was undertaken by considering clustering pairs which contained at least one case from a more densely populated area (‘more densely populated: any’) and clustering pairs which contained at least one case from a less densely populated area (‘less densely populated: any’). The methods are given in more detail in Birch *et al* (2000) and Mcnally *et al* (2002).

## RESULTS

The study included 512 cases of ALL, comprising 395 precursor B-cell ALL, 63 T-cell, 36 Null-ALL, 9 B-cell, 1 non-B, non-T cell and eight unclassified. Based on place and time of birth the only statistically significant evidence of space-time clustering was for cases of precursor B-cell ALL, aged 18–54 months (*P*=0.05, using the NN threshold version of the K-function method, Table 1a

**Table 1:**
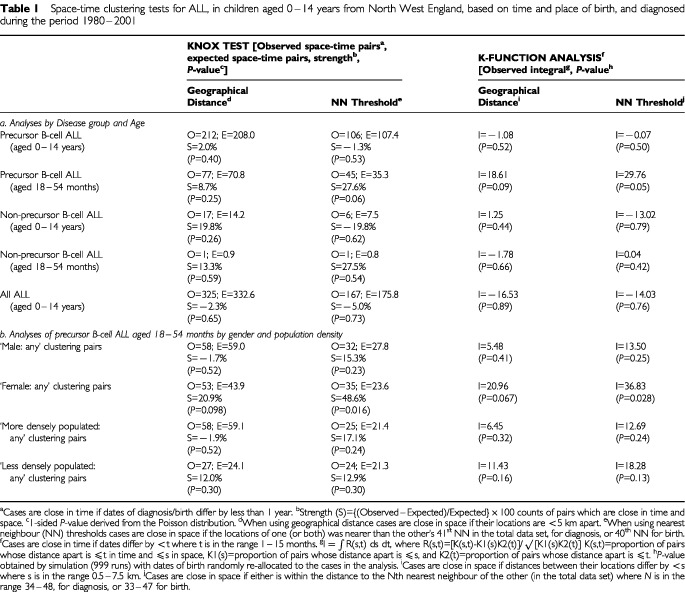
Space-time clustering tests for ALL, in children aged 0–14 years from North West England, based on time and place of birth, and diagnosed during the period 1980–2001

). Examination of cases of precursor B-cell ALL, aged 18–54 months, by gender and population density (Table 1b) shows that the space-time clustering was confined to clustering pairs of cases which contained at least one female case (*P*<0.1, using all four methods). There was no association with population density level. There was no evidence for space-time clustering in any group based on time and place of diagnosis, time of diagnosis/place of birth nor on time of birth/place of diagnosis.

## DISCUSSION

Our previous study (Birch *et al*, 2000) showed striking space-time clustering among a proportion of cases of ALL aged 0–4 years, which was based on time of diagnosis/place of birth. In contrast, the present study found limited evidence for clustering based on time of birth/place of birth only. However, even though the results were statistically less significant than in the previous larger study, the clustering for the 18–54 month age group (the childhood peak) of precursor B-cell ALL clearly stood out. This is the first study to demonstrate that space-time clustering for cases of ALL in the childhood incidence peak is specifically due to the precursor B-cell sub-type. The results are consistent with a role for infections, but with exposure occurring pre-natally, or around the time of birth.

The clustering was only apparent among space-time pairs that involved at least one female case. This differs from our previous study, which found an excess of male cases over females involved in space-time pairs. Interestingly, our recent study of incidence trends in ALL (Mcnally *et al*, 2000), showed that the observed increase was attributable to the precursor B-cell sub-type, and indicated a faster recent rate of increase amongst females.

The present data appear to be more consistent with Smith's hypothesis (Smith, 1997) that the childhood peak is due to an in-utero exposure to infection, rather than with Greaves’ hypothesis of delayed exposure to common infections (Greaves, 1988). The data are also consistent with Kinlen's hypothesis of population mixing (Kinlen, 1995), particularly in relation to one study which demonstrated a marked excess of leukaemia in children under 1 year of age suggesting an infection during pregnancy (Kinlen and Hudson, 1991). The differences between males and females may be indicative of an immune modulation hypothesis, with females being more susceptible to leukaemogenic events resulting from exposure to infection. Relative to our earlier findings the current evidence would suggest that there is greater exposure to the aetiological agent at an earlier age, or a greater prevalence of the agent, or a new aetiological agent. Whichever of these applies we would hypothesise that the agent would be an infection. Finally, it should be stressed that a direct comparison between this study and the previous one is not possible, because data on immunophenotype were not available in the previous analysis.
